# Benzodiazepine Overuse in Elders: Defining the Problem and Potential Solutions

**DOI:** 10.7759/cureus.11042

**Published:** 2020-10-19

**Authors:** Todd Gress, Mark Miller, Charles Meadows, Shirley M Neitch

**Affiliations:** 1 Internal Medicine, Joan C. Edwards School of Medicine, Marshall University, Huntington, USA; 2 Psychiatry, West Virginia University School of Medicine, Morgantown, USA

**Keywords:** prevalence, benzodiazepines, geriatrics, appalachian region

## Abstract

Objective

To determine the prevalence of benzodiazepine use in adults aged 65 and older at two West Virginia academic medical centers as phase one of a benzodiazepine deprescribing strategy.

Design

Cross-sectional

Setting

Two academic hospitals in West Virginia with 107,504 hospitalized adults age 65 and older from the years 2010 to 2018 with information on admission medication use.

Measurements

Use of benzodiazepines based on presence on the admission medication list. Demographics, select co-morbidities, and laboratory tests were also recorded.

Results

The prevalence of benzodiazepine use was 13.5% and use remained relatively constant with increasing age over 65, even in those over age 89.

Conclusion

Efforts aimed at assessing the true need for benzodiazepine use and deprescribing need to be employed, particularly with advancing age.

## Introduction

While the nation has appropriately focused on the opioid overprescribing crisis, benzodiazepine overuse is another class of potentially harmful medication that has quietly become pervasively problematic. Benzodiazepines have a less deadly profile than opioids but can cause numerous serious problems, especially among the older population. Despite these problems, benzodiazepine prescriptions written in primary care settings continue to rise dramatically [1].

Rational pharmacotherapy for older patients takes multiple factors into account, including comorbidities, polypharmacy, fitness versus frailty, compliance, age-dependent pharmacokinetics/pharmacodynamics, and drug-drug interactions [2-5]. Known dangers of benzodiazepines for older patients include lethargy, increased confusion, increased risk of falls and fractures, significant impairment of driving skills with increased crash risk, and increased risk of an emergency room visit [6-11]. Long-term benzodiazepine use fosters dependence and exposure to potentially serious consequences, such as withdrawal-induced delirium, seizures, and death. There is also evidence, albeit somewhat conflicting, that long-term use of benzodiazepines will increase the risk of dementia [12-13]. The above risk factors can be further exacerbated by the combined use of other drugs, particularly alcohol and opiates. For these reasons, benzodiazepines have been on the Beers List® of suggested drugs to avoid in older people since its first iterations in the 1990s, and they remain on the newest update to the list in 2019 [14].

Despite these risks, recent studies in adults have demonstrated nearly a doubling of primary care office visits nationally between 2003 and 2015 in which a benzodiazepine was prescribed (3.8% to 7.4%) [15] and a high frequency (29.5%) of hospitalized patients receiving at least one benzodiazepine during hospitalization [16].

Older adults in general are quite willing to allow their doctor to deprescribe other medications to simplify their medication regimen or to cut costs, but agreeing to cut benzodiazepines is often met with reluctance. Some of the reasons older patients state they resist a reduction in benzodiazepine use include fear of a return of anxiety that is perceived to be well-controlled by long-term benzodiazepine use, lack of perceived harm, fear of worsening insomnia, and the perceived need for continued access to benzodiazepines to cope with anxiety-provoking social situations. Reasons for failure in attempting to deprescribe benzodiazepines in a study of 261 older chronic users [17] included a lack of support from a healthcare provider, a focus on short-term quality of life, intolerance to withdrawal symptoms, and perceived poor health. Reducing the prevalence of use in elders, therefore, requires a two-pronged approach: 1) reducing new prescriptions for benzodiazepines going forward through education and regulatory oversight of prescribers, and 2) deprescribing current benzodiazepine usage. Deprescribing requires a long-term sustained effort to educate consumers and providers about the dangers of benzodiazepine use with advancing age, the availability of suitable alternative strategies for coping with anxiety and insomnia, and the reassurance that the deprescribing process will be carried out cautiously at a pace they can tolerate with adequate ongoing support during this time-dependent process. This latter point is often missing from many prescribers' attempts to deprescribe benzodiazepines because they misperceive the intensity of the heightened anxiety in the patients who perceive they are dependent upon them for normal function. Furthermore, intervals between deprescribing follow-up visits often leave the patient feeling alone with their fears of overwhelming anxiety or insomnia without adequate back-up. Deprescribing must be seen as a process, not a discrete event, with allowances made for more frequent follow-up encounters, whether this is in person, by phone, or virtually. 

Educating prescribers about the appropriate use of benzodiazepines in clinical practice should begin in medical school and be augmented by continuing education throughout the prescriber’s career. Some health system formularies track benzodiazepine prescriptions for elders as part of physician quality performance evaluations. These programs act as appropriate disincentives for prescribing benzodiazepines for elders and incentives for deprescribing. Systematic deprescribing efforts that have also been studied with reasonable success rates, such as in the Eliminating Medications Through Patient Ownership of End Results (EMPOWER) study [18-20], require sustained psycho-education for consumers over a period of months to succeed in winning the trust of the patient. This is coupled with offers of alternative strategies, such as SSRI use for anxiety control, improved sleep hygiene, and safer medication strategies for insomnia. Successful deprescribing requires that physicians adopt a long-term sustained effort that is often difficult to achieve in a typical office practice. This may leave physicians frustrated and nihilistic about the chances of achieving the desired clinical outcomes. Success can be jeopardized by taking an abrupt stance of too rapid discontinuation or refusal to refill benzodiazepine prescriptions. These approaches often backfire when patients show withdrawal complications or consumers revert to access benzodiazepines in other ways as the critical buy-in to the deprescribing process was never achieved. 

Recent data show a higher prevalence of benzodiazepine use in the Appalachian region compared to the national average. The Appalachian region also has a higher proportion of older residents compared to the general population [21]. For these reasons, intervention strategies intended to reduce benzodiazepine usage are particularly important. Marshall University and West Virginia University, taken together, are geographically representative of the entire state of West Virginia. Therefore, the two institutions undertook a collaborative effort with grant support from the Claude Worthington Benedum Foundation and the West Virginia Higher Education Policy Commission to forge a two-pronged effort to 1) further delineate the extent of benzodiazepine use and 2) to develop and test strategies for improving quality of life by reducing benzodiazepine usage in elders. In order to ascertain if intensified deprescribing programs in our region are warranted, we began by analyzing data from hospitalized adults aged 65 years and older and contrasted this data with comparative national rates.

## Materials and methods

We evaluated a cross-section of all adults aged 65 years and over from their first admission to our academic hospitals (n: 107,504) in Huntington and Morgantown, West Virginia from the years 2010 to 2018. All data were de-identified and obtained from each respective institution’s data warehouse. Our study was approved by the Marshall University Institutional Review Board.

We collected demographic information, including age, gender, and insurance type (none, Medicare, Medicaid, and private). Patients over 89 years of age were assigned the average age for that age group to protect their identity. We collected information on the following diagnoses: dementia, Alzheimer’s dementia, diabetes, and hypertension. We additionally collected laboratory information on serum albumin (gm/dL), hemoglobin (gm/dL), and serum creatinine (gm/dL) as additional measures of health status. We recorded total hospital charges, hospital length of stay, whether or not the patient required the intensive care unit (ICU), and in-hospital mortality.

Benzodiazepine use upon hospital admission was considered to be present if any of the following benzodiazepines were identified on the home medication list: alprazolam, chlordiazepoxide, clonazepam, clorazepate, diazepam, flurazepam, lorazepam, oxazepam, quazepam, temazepam, or triazolam. Benzodiazepine use was grouped together and recorded dichotomously as either present or absent on the home medication list.

All variables were described using simple descriptive statistics. We assessed the normality of our continuous variables graphically and with the Shapiro Wilk test. We compared all study variables for those with and without benzodiazepine use on admission using chi-square for categorical variables and the Student’s t-test for continuous variables (Wilcoxon Rank Sum if non-normal distribution). All other comparisons were made using the same statistics. We adjusted mortality rates for age and gender using multiple logistic regression. We evaluated the prevalence of benzodiazepine use by age category using multiple logistic regression with the lowest age category as the referent. All analyses were performed using the Stata software, version 16.0 (StataCorp LLC, College Station, Texas, USA).

## Results

We identified 107,504 unique patients aged 65 years and over admitted to our hospitals from 2010 to 2018 with admission medication history available. Patients were an average of 75.5 years old and the majority were female (Table 1). The prevalence of diabetes (41.2%), hypertension (86.6%), and dementia (28.2%) were what was expected for this population. A specific diagnosis of Alzheimer’s dementia was present in 3.8%. The mean values of serum creatinine, hemoglobin, and albumin were within the normal range. A total of 11.7% of patients required an ICU stay during their admission. Finally, 13.5% of all patients reported home use of a benzodiazepine.

**Table 1:** Baseline Characteristics of 107,504 Hospitalized Adults Age 65 Years and Older Numbers in parentheses represent the total N unless otherwise indicated IQR: interquartile range; SD: standard deviation;

Characteristic	N = 107,504
Age, mean years (SD)	75.50 (7.66)
Gender, % male	44.84 (48,200)
Insurance Type	
Medicare, % (n)	77.74 (83,573)
Private, % (n)	21.14 (22,723)
Medicaid, % (n)	0.58 (624)
None, % (n)	0.54 (584)
Diabetes, % (n)	41.18 (44,274)
Hypertension, % (n)	86.62 (93,118)
Dementia, % (n)	28.24 (30,357)
Alzheimer’s, % (n)	3.75 (4,033)
Serum creatinine (gm/dL), mean (SD)	1.35 (1.18)
Serum hemoglobin (gm/dL), mean (SD)	12.14 (2.22)
Serum albumin (gm/dL), mean (SD)	3.34 (0.71)
Length of stay, median days, (IQR)	3.0 (4.0)
Total hospital charges, median (IQR)	$34,156 ($41,800)
Intensive care unit, % (n)	11.70 (12,575)
In-hospital mortality, % (n)	4.20 (4,519)
Benzodiazepine on admission, % (n)	13.46 (14,467)

We compared all study variables by reported home use of a benzodiazepine (Table 2). Those with benzodiazepine use were slightly younger (75.24 benzodiazepine vs. 75.54 years no benzodiazepine; p < 0.001) and more likely to be female (65.4% benzodiazepine vs. 53.6% no benzodiazepine; p < 0.001). Home use of a benzodiazepine was significantly different for those with diabetes and hypertension, although these differences were small. Benzodiazepine use was significantly higher in those with dementia (33.5% vs. 27.4%; p < 0.001) and Alzheimer’s (5.5% vs. 3.5%; p < 0.001) compared to those without dementia and Alzheimer's (all p < 0.001). There were minor clinical differences in the mean values of serum hemoglobin, creatinine, and albumin, although these differences were statistically different due to the large sample size. Median length of stay (4.0 vs. 3.0 days; p < 0.001) and median total hospital charges ($34,689 vs. $34,080; p = 0.03) were higher in those with reported home benzodiazepine use. An admission requiring an ICU stay was higher in those with benzodiazepine use (12.5 vs. 11.6%; p = 0.001). Finally, crude in-hospital mortality was slightly lower for those with reported home use of benzodiazepines (3.8 vs. 4.3%; p = 0.006). However, when in-hospital mortality rates were adjusted for age and gender, the rates were similar for those with and without benzodiazepine use (3.9 vs. 4.2%; p = 0.11).

**Table 2:** Characteristics of 107,504 Hospitalized Adults Age 65 and Older by Reported Home Benzodiazepine Use *P-value across categories Numbers in parentheses represent the total number (N) unless otherwise indicated IQR: interquartile range; SD: standard deviation

Characteristic	Benzodiazepine (N = 14,467)	No Benzodiazepine (N = 93,037)	P-value
Age, mean years (SD)	75.24 (7.60)	75.54 (7.67)	< 0.001
Gender			
Female, % (n)	65.39 (9,460)	53.57 (49,844)	< 0.001*
Male, % (n)	34.61 (5,007)	46.43 (43,193)	
Insurance			
Medicare, % (n)	80.58 (11,657)	77.30 (71,916)	< 0.001*
Private, % (n)	18.55 (2,683)	21.54 (20,040)	
Medicaid, % (n)	0.47 (68)	0.60 (556)	
None, % (n)	0.41 (59)	0.56 (525)	
Diabetes, % (n)	39.83 (5,762)	41.39 (38,512)	< 0.001
Hypertension, % (n)	87.29 (12,628)	86.51 (80,490)	0.01
Dementia, % (n)	33.48 (4,844)	27.42 (25,513)	< 0.001
Alzheimer’s, % (n)	5.50 (795)	3.48 (3,238)	< 0.001
Serum creatinine (gm/dL), mean (SD)	1.29 (1.10)	1.36 (1.19)	< 0.001
Serum hemoglobin (gm/dL), mean (SD)	12.02 (2.15)	12.16 (2.23)	< 0.001
Serum albumin (gm/dL), mean (SD)	3.39 (0.69)	3.33 (0.71)	< 0.001
Length of Stay, median days (IQR)	4.0 (5.0)	3.0 (4.0)	< 0.001
Total hospital charges, median dollars (IQR)	$34,689 ($44,043)	$34,080 ($41,539)	0.03
Intensive care unit, % (n)	12.5 (1,809)	11.6 (10,766)	0.001
In-hospital mortality, % (n)	3.78 (547)	4.27 (3,972)	0.006

**Figure 1:**
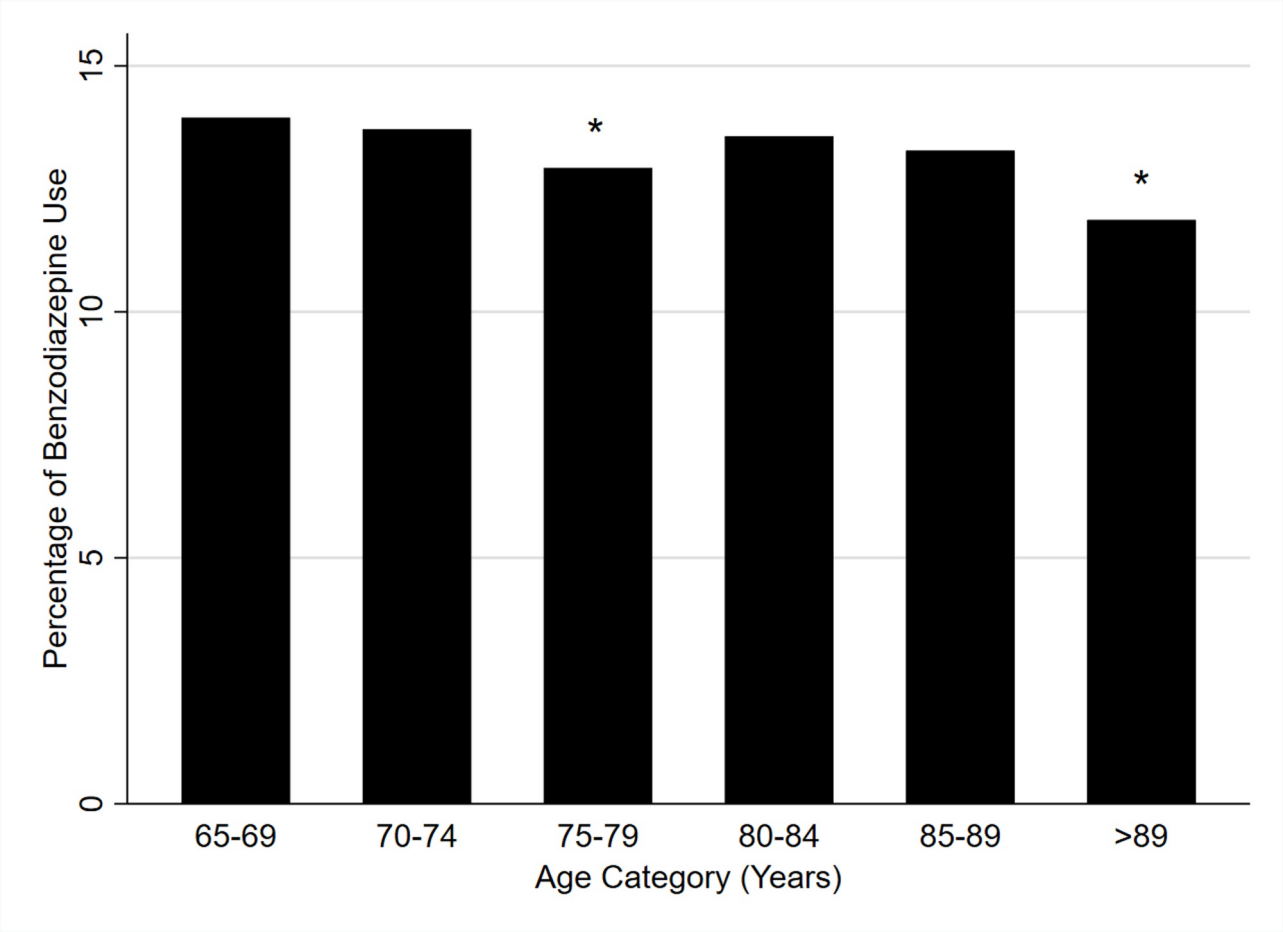
Percent Reported Home Benzodiazepine Use by Age Category Among Hospitalized Adults Age 65 Years and Older *p < 0.01 for comparison to age category 65 - 69 years

We examined the effects of age on reported home use of benzodiazepines (Figure 1). Although there are some subtle differences in percent benzodiazepine use when comparing the older age categories to the baseline category of 65 - 69, for the most part, use was not much different by age category with reported use over 10% for each age category.

## Discussion

We found that the prevalence of benzodiazepine use, based on reported home medications at the time of admission in hospitalized adults aged 65 years and older, was 13.5%. Women and those with dementia, including Alzheimer’s dementia, were much more likely to report the use of a benzodiazepine. Reported benzodiazepine use on admission was associated with a longer length of stay and a higher likelihood of admission to the ICU. Importantly, and unfortunately, the prevalence of benzodiazepine use did not decline with increasing age.

This study demonstrated the linkage of benzodiazepine use with multiple factors, many of which are known, but two of these, in particular, seem to demand a closer look. The first is the association of benzodiazepine use with dementia and Alzheimer’s dementia, in particular. Other studies have shown similar findings with the association being strongest with long-term benzodiazepine use [12-13, 22]. Although several mechanisms for such an effect have been proposed, there is no specific evidence for causality. It may simply be that patients with dementia are perceived by some providers to need the potent sedative and anxiolytic properties of benzodiazepines. 

The second factor which begs attention is the high overall use rate of benzodiazepines. Our overall prevalence rate (13.5%) is consistent with other studies [16, 23-25]. Similar studies have demonstrated that benzodiazepine use is increasing and that a large number of older patients receive benzodiazepine during hospitalization [15, 26]. This is particularly concerning given that new benzodiazepine prescriptions given to older patients at hospital discharge may lead to chronic benzodiazepine use [27]. Furthermore, we found that the prevalence of benzodiazepine use did not decline with advancing age over 65. Even those in the highest age category and the most vulnerable to the effects of a sedative-hypnotic had a reported prevalence of 11.9% for benzodiazepine use.

We did not find a higher incidence of benzodiazepine prescribing in this Appalachian population when compared to previously reported studies of diverse populations. While the region has been fraught with the problems associated with the overuse of opioids, alone and in combination with other drugs (which are often benzodiazepines), the actual prescribing of these medications seem to be on par with other regions. 

Our study supports previously described associations and corroborates some concerning trends with benzodiazepine use in older adults. Many previous studies have demonstrated numerous adverse effects of this class of drugs in older adults, including a potential link to dementia [4-13]. Given these demonstrated associations of adverse effects, there is at least some concern that we may be causing harm by prescribing benzodiazepines. Therefore, efforts should be aimed at assessing the true need for benzodiazepine use and deprescribing when possible, particularly in the older patient population.

There are several limitations to our study. First and foremost, this is a cross-sectional study, and any associations identified are based upon the prevalence of benzodiazepine use. However, determining the prevalence of benzodiazepine use was the major goal of our study. Second, we did not try to examine the type of benzodiazepine or the dose of the benzodiazepine, but rather we looked at benzodiazepines as a class of drugs since our focus was on the prevalence of the use of this drug class in older adults. Third, we did not attempt to verify the admission medication list, and therefore, potential reporting errors could have occurred. However, we believe most errors on the admission medication identification would be that of omission [28], which would likely result in underreporting of benzodiazepine use; therefore, the prevalence rate that we identified would be at least at the minimum. Fourth, our patient population was from hospitalized older adults in the Appalachian region and, therefore, may not be generalizable. Also, hospitalized patients may represent a subpopulation more likely to be prescribed a benzodiazepine. Finally, we utilized a large dataset from data warehouses for our study, and analyses of large datasets are associated with several issues, such as overinterpretation of results based on p-values instead of effect size [29]. However, we were careful to point out important differences based more on effect size because most of our differences were significant at a two-tailed probability of less than 5%.

## Conclusions

We found that benzodiazepine use in the Appalachian region is similar to national rates of benzodiazepine use and that prevalence rates of use remain about the same with advancing age. Given the known adverse effects of this drug class, particularly in the elderly, efforts should be aimed at a rigorous assessment of the true need for a benzodiazepine, along with the implementation of deprescribing practices for existing benzodiazepine users.
